# Impact of ototoxic agents and noise exposure on hearing loss among healthcare personnel in a medical university: a cross-sectional analytical study

**DOI:** 10.1186/s12889-025-25371-8

**Published:** 2025-11-20

**Authors:** Thitiworn Choosong, Sasithorn Srimeechai, Ramida Dindamrongkul, Wandee Khaimook

**Affiliations:** 1 Faculty of Medicine, Prince of Songkla University, Hat Yai, Songkhla Thailand; 2 Faculty of Public Health, Thammasat University, Pathum Thani, Thailand

**Keywords:** Antineoplastic drug, Healthcare worker, Hearing loss, Noise exposure, Ototoxic agent

## Abstract

**Background:**

Ototoxic agents, such as xylene, formaldehyde, mercury, and antineoplastic agents, are routinely used in hospitals. Studies on hearing loss (HL) among healthcare workers (HCWs) exposed to ototoxic agents and noise remain limited in Thailand. In this study, we aimed to investigate the combined effects of low-level occupational noise and ototoxic agent exposure and evaluate the prevalence of HL among HCWs.

**Methods:**

This cross-sectional analytical study was conducted at a university hospital in Thailand between December 2022 and October 2023. Ototoxic exposure was assessed using self-reported data verified by the researchers. Occupational noise exposure levels were measured using a dosimeter, and HL was evaluated using audiometry. Multiple regression analysis was performed to identify factors associated with HL.

**Results:**

Of 169 participants who underwent audiometric tests, 17.2% (29/169 participants) had HL, with 20.3% (16/79 exposed) in the exposed group and 14.4% (13/90 unexposed) in the non-exposed group (*p* > 0.05). HCWs exposed to antineoplastic drugs and those working in the pharmacy department demonstrated the highest mean noise levels (75.20 ± 3.94 dBA and 77.68 ± 3.81 dBA, respectively). Furthermore, the average number of elevated unweighted peak noise events was observed in the pharmacy (232.89 ± 88.66 events) and emergency (230.96 ± 60.56 events) departments. Age, sex, and obesity were significantly associated with HL.

**Conclusions:**

A high prevalence of HL was observed among HCWs despite low noise exposure levels, underscoring the substantial impact of combined exposure to occupational noise and ototoxic agents. The identification of groups that are particularly vulnerable to antineoplastic drugs, such as HCWs, guides targeted preventive measures and further research in this area.

**Supplementary Information:**

The online version contains supplementary material available at 10.1186/s12889-025-25371-8.

## Background

Noise-induced hearing loss (NIHL) resulting from occupational noise exposure is well recognised, with varying prevalence rates among workers in different occupations exposed to loud noises [1, 2]. In Thailand, studies conducted between 1998 and 2023 reported an NIHL prevalence of 21.2–53.8% in hospital settings [3–9]. These studies included departments, such as central sterile supply, laundry, nutrition and dietetics, plaster rooms, dental clinics, engineering, maintenance, and utilities, where average noise levels exceeded the national standard in Thailand and the American Conference of Governmental Industrial Hygienists (ACGIH) occupational exposure limit (OEL) of 85 dBA [3–9]. Additionally, the prevalence of NIHL has been documented in intensive care units, paediatric intensive care units, and operating rooms (ORs) worldwide, despite average noise levels remaining below 85 dBA [10–14].

Reports on the prevalence of HL among healthcare workers (HCWs) exposed to low-level noise (< 85 dBA for an 8-h working day) in Thailand are currently lacking. This gap highlights the need to examine the contribution of factors other than acoustic exposure, such as ototoxic agents, to HL among HCWs in Thailand. Co-exposure to noise and ototoxic agents [15]—including antineoplastic medications (e.g. cisplatin), solvents (e.g. toluene, p-xylene), formaldehyde and metals (e.g. lead, mercury)—commonly occurs in hospital settings. Nevertheless, studies on HL among HCWs exposed to ototoxic agents and noise remain limited, particularly in Thailand.

Addressing occupational HL, particularly in hospital environments, is crucial for improving occupational health and safety. Therefore, we aimed to characterise low-level noise exposure and assess the prevalence of HL among HCWs at a medical university with particular attention to noise and ototoxic agents as potential contributing factors.

## Methods

This cross-sectional analytical study was conducted among HCWs at a medical school in Hat Yai District, Songkhla, Thailand, from December 2022 to October 2023. The purposive sampling technique was used to recruit HCWs who volunteered to participate in the study and were exposed to ototoxic agents and worked in noisy environments. Written informed consent was obtained from all participants in accordance with the Belmont Report guidelines. The study was approved by the Human Research Ethics Committee of the university (REC. 65–444-9-2).

### Participants and study setting

The study population was recruited based on job characteristic, work area, and exposure to ototoxic agents. Work areas, such as the internal medicine respiratory unit (MRCU), emergency room (ER), and OR, were categorised as ‘non-ototoxic agent areas’. Contrarily, departments, such as the antineoplastic outpatient clinic, pharmacy (specifically those involved in the preparation of antineoplastic drugs), physical pathology, and dental departments, were classified as ‘ototoxic agent areas’.

Exposure to ototoxic agents was initially self-reported by participants and subsequently verified by the researcher. Exposure classification was determined based on job characteristics, previous environmental monitoring data, and on-site inspections. The categories of ototoxic exposure were as follows: (1) non-exposed, (2) xylene, (3) formaldehyde, (4) mercury, (5) antineoplastic drugs, and (6) combined exposure to xylene and formaldehyde. The non-exposure category referred to either the absence of these agents in the work areas or a negligible risk of job-related exposure, such as the handling of small tissue samples sealed in formaldehyde by OR staff. Staff in the MRCU who were assigned to patients undergoing antineoplastic treatment were categorised as having antineoplastic drug exposure. All dental staff, excluding administrative personnel, were assigned to the mercury exposure group due to amalgam use, even in small amounts.

Participants in the chemotherapy outpatient, pharmacy, physical pathology, and ER departments provided self-reported exposure data that were consistent with the researchers’ professional assessment. However, instances of misclassification bias were noted, where participants perceived ototoxic exposure despite insufficient evidence or inadequate information regarding workplace conditions.

HCWs from the specified departments were included in the study. Written consent forms and self-report questionnaires were obtained from each participant, followed by a physical ear examination conducted by an otolaryngologist and the researcher. HCWs with a history of tinnitus, ruptured eardrums, active ear disease, or current or past chemotherapy were excluded. Participants were ultimately reclassified into (1) non-exposure, (2) antineoplastic exposure, and (3) other ototoxic agent exposure groups.

### Data collection and instruments

A self-administered questionnaire was developed (Supplementary File) and used to obtain general and occupational information from the participants, including age, sex, weight, height, work history, noise exposure history, current and past medication use (e.g. antibiotics, loop diuretics, analgesic drugs such as nonsteroidal anti-inflammatory drugs and antineoplastic drugs), ototoxic agent exposure, tobacco smoking, alcohol and coffee consumption, and hearing protective device [HPD] use.

### Noise exposure measurement

Noise exposure levels were monitored during working hours using a Lason Davis noise dosimeter attached to the collars of HCWs. The monitor was set with an 80-dBA threshold level, an 85-dBA criterion level, a 3-dB exchange rate, and an 8-h criterion time with measurements integrated over a range of 80–140 dB. Calibration checks were performed before and after each monitoring session. The primary metrics recorded were the 8-h time-weighted average (TWA, dBA), maximum noise level (Lmax, dBA), peak noise level (Lpeak, dB), and the number of noise peaks detected during the sampling period.

### Audiometric testing

All participants underwent standard pure tone audiometry (PTA) assessment (ANSI/ASA S3.1–1999) in a soundproof room. Pure tone stimuli at frequencies ranging from 0.25 to 8 kHz were administered using headphones. The participants were instructed to indicate sound perception by pressing a button. Hearing thresholds were determined using the Hughson-Westlake procedure. As baseline audiometric data were not available, current audiograms were used to assess HL. The participants with average hearing thresholds >25 dB at 1, 2, 3 and 4 kHz were diagnosed with HL [16, 17].

### Data analysis

Descriptive statistics, such as frequencies, percentages, means, and standard deviations, were used to summarise the general characteristics of the participants, noise exposure levels, and the prevalence of HL. Obesity was defined according to the Asia-Pacific region body mass index (BMI) criteria of the World Health Organization, International Association for the Study of Obesity, and International Obesity Task Force [18], as a BMI >25 kg/m^2^. Inferential statistics, including the chi-square test, Fisher’s exact test, and Wilcoxon signed-rank test, were used to assess the associations between variables (*P* < 0.05). Multiple logistic regression analysis was performed to identify factors associated with HL. In the initial model, variables with p-values ≤ 0.2 from the univariable analysis were included. The backward selection method was then applied to confirm the associated factors.

## Results

In total, 170 participants were classified into the non-exposure, antineoplastic exposure, and other ototoxic agent exposure groups. Among the surveyed factors, analgesic use showed the highest prevalence (50.7%) in the non-exposure group, whereas HPD use was extremely rare across all groups (Table 1).

**Table 1:** General characteristics of participants classified by ototoxic agent exposure (self-assessment questionnaire) (n = 170)

Parameter	Exposure			*P* value
	Non-exposure (n = 90)	Antineoplastic (n = 48)	Other ototoxic agents (n = 32)	*Chi-square test*
Age (years)				<.001*
Mean ± sd	37.72 ± 9.27	41.81 ± 9.04	39.16 ± 10.34	
min–max	24–59	24–59	24–59	
Sex				0.05**
Male (n = 12)	3 (25.0)	4 (33.3)	5 (41.7)	
Female (n = 158)	87 (55.1)	44 (27.8)	27 (17.1)	
Marital Status				0.13**
Single (n = 73)	42 (57.5)	17 (23.3)	14 (19.2)	
Married (n = 86)	46 (53.5)	25 (29.1)	15 (17.4)	
Divided (n = 11)	2 (18.2)	6 (54.5)	3 (27.3)	
Obesity				0.18
Non-obesity (n = 127)	65 (51.2)	34 (26.8)	28 (22.0)	
Obesity (n = 43)	25 (58.1)	14 (32.6)	4 (9.3)	
Work Experience (years)				<.001*
Mean ± sd	12.59 ± 10.18	12.77 ± 9.15	11.00 ± 9.00	
min–max	1–40	1–39	1–33	
Working hour (hours)	8.41 ± 1.66	8.83 ± 2.98	7.00 ± 2.62	<.001*
Past Exposure to Noise				<.001
No (n = 126)	74 (58.7)	26 (20.6)	26 (20.6)	
Yes (n = 39)	12 (30.8)	22 (56.4)	5 (12.8)	
HPD using				0.69**
No (n = 168)	88 (52.4)	48 (28.6)	32 (19.0)	
Yes (n = 2)	2 (100)	0	0	
Earbud using				<.001
Yes (n = 88)	47 (53.4)	27 (30.7)	14 (15.9)	
No (n = 82)	43 (52.4)	21 (25.6)	18 (22.0)	
Alcohol consumption				0.14**
Yes (n = 35)	15 (42.9)	9 (25.7)	11 (31.4)	
Ex-drinker (n = 9)	4 (44.4)	2 (22.2)	3 (33.3)	
Never (n = 126)	71 (56.3)	37 (29.4)	18 (14.3)	
Coffee consumption				0.14**
Yes (n = 109)	52 (47.7)	33 (30.3)	24 (22.0)	
Ex-drinker (n = 6)	2 (33.3)	3 (50.0)	1 (16.7)	
Never (n = 55)	36 (65.5)	12 (21.8)	7 (12.7)	
Tobacco smoking				0.01**
Never (n = 167)	90 (53.9)	48 (28.7)	29 (17.4)	
Ex-smoker (n = 2)	0	0	2 (100)	
Current smoker (n = 1)	0	0	1 (100)	
Antibiotics drugs (aminoglycosides, ampicillin, macrolides)				0.81**
No (n = 165)	88 (53.3)	46 (27.9)	31 (18.8)	
Yes (n = 5)	2 (40.0)	2 (40.0)	1 (20.0)	
Loop diuretics				0.07**
No (n = 167)	90 (53.9)	47 (28.1)	30 (18.0)	
Yes (n = 3)	0	1 (33.3)	2 (66.7)	
Analgesics (aspirin/salicylates, paracetamol, codeine, indomethacin, ibuprofen, phenylbutazone)				0.88
No (n = 101)	55 (54.5)	28 (27.7)	18 (17.8)	
Yes (n = 69)	35 (50.7)	20 (29.0)	14 (20.3)	

*HPD* Hearing protective devices

*Wilcoxon signed-rank test

**Fisher's exact test

The general characteristics of participants—including age, work experience, working hours, history of noise exposure, earbud use, tobacco smoking, and medication use—varied significantly among participants in the exposure groups. HCWs in the pharmacy department demonstrated the highest 8-h TWA noise exposure (77.68 ± 3.81 dBA), whereas those in physical pathology were exposed to the lowest levels (70.59 ± 3.24 dBA) (Table 2).

**Table 2:** Ototoxic agents and noise exposure classified by working area (n = 170)

Parameters	Working area								*P* value*
	Operation room		Chemotherapy OPD (n = 23)	Pharmacy department (n = 18)	Dentistry department (n = 19)	Emergency department (n = 19)	Internal medical respiratory ICU (n = 13)	Physical pathology department (n=18)	
	Anaesthesia staff (n = 31)	Scrub staff (n = 29)							
Noise Exposure									
8-h TWA, dBA	72.18 ± 5.5	71.43 ± 4.77	73.5 ± 3.52	77.68 ± 3.81	71.47 ± 5.48	72.81 ± 3.90	74.51 ± 3.00	70.59 ± 3.24	<.001
Lmax, dBA	98.11 ± 5.21	96.81 ± 4.90	97.64 ± 3.40	102.76 ± 5.17	98.68 ± 5.77	98.05 ± 4.19	97.99 ± 3.62	96.81 ± 4.14	<.001
Lpeak, dB	131.15 ± 6.53	128.99 ± 5.56	127.06 ± 4.28	130.34 ± 5.80	127.77 ± 5.21	136.9 ± 17.32	127.30 ± 6.63	132.2 ± 14.52	<.001
Number of Lpeak	185.34 ± 54.42	178.61 ± 61	170.96 ± 59.92	232.89 ± 88.66	132.11 ± 72.39	230.96 ± 60.56	176.79 ± 76.57	158.06 ± 49.30	<.001

*ICU* Intensive care unit, *OPD* Outpatient department, *TWA* Time-weighted average

* Wilcoxon signed-rank test

When analysed by ototoxic exposure category, the highest 8-h TWA noise levels were observed among HCWs exposed to antineoplastic drugs (75.20 ± 3.94 dBA), whereas the lowest levels were recorded among those exposed to xylene (69.9 ± 3.15 dBA) (Table 3). Notably, 8-h TWA noise levels across all departments remained below the OEL of 85 dBA.

**Table 3:** Noise characteristics classified by ototoxic agent exposure (*n* = 170)

Parameter	Exposure							*P* value*
Noise Exposure	Non-exposure (*n* = 90)	Antineoplastic drug (*n* = 48)	Other ototoxic agents					
			Total (*n* = 32)	Xylene (*n* = 6)	Formaldehyde (*n* = 5)	Mercury (*n* = 14)	Xylene & Formaldehyde(*n* = 7)	
8-hour TWA, dBA	71.91 ± 5.02	75.20 ± 3.94	71.54 ± 4.19	69.9 ± 3.15	71.86 ± 1.58	72.76 ± 5.02	70.29 ± 4.23	< 0.001
Lmax, dBA	97.46 ± 4.77	99.52 ± 4.71	98.04 ± 5.17	94.8 ± 3.31	96.82 ± 2.33	99.61 ± 6.05	98.53 ± 5.32	< 0.001
Lpeak, dB	131.42 ± 10.55	127.88 ± 5.17	130.53 ± 11.48	138.97 ± 24.08	128.78 ± 5.90	128.37 ± 5.46	128.84 ± 4.74	< 0.001
Number of Lpeak	188.79 ± 71.56	195.23 ± 76.54	150.00 ± 58.63	145.83 ± 54.55	163.00 ± 60.51	139.64 ± 69.37	165.00 ± 41.71	< 0.001

*Wilcoxon signed-rank test was usded to determine the significant difference between

1) non-exposure, antineoplastic, and other ototoxic agents and

2) non-exposure, antineoplastic, xylene, formaldehyde, mercury, and xylene and formaldehyde groups

Overall, 169 participants underwent audiometric testing; conventional PTA revealed an HL prevalence of 17.2%, comprising 9.5% unilateral and 7.7% bilateral HL cases. Regarding hearing abnormalities, 16.7% of HCWs exposed to antineoplastic agents and 25.8% exposed to other ototoxic agents were affected (Table 4).

**Table 4:** Hearing abnormality of participants classified by exposure groups (*n* = 169)

Parameter	Total	Exposure			*P* value fisher’s exact test
		Non-exposure (*n* = 90, %)	Antineoplastic (*n* = 48, %)	Other ototoxic agents (*n* = 31, %)	
Normal	140 (82.8)	77 (85.6)	40 (83.3)	23 (74.2)	0.375
Abnormal	29 (17.2)	13 (14.4)	8 (16.7)	8 (25.8)	
- Unilateral	16	8	6	2	
- Bilateral	13	5	2	6	

**Fig. 1:**
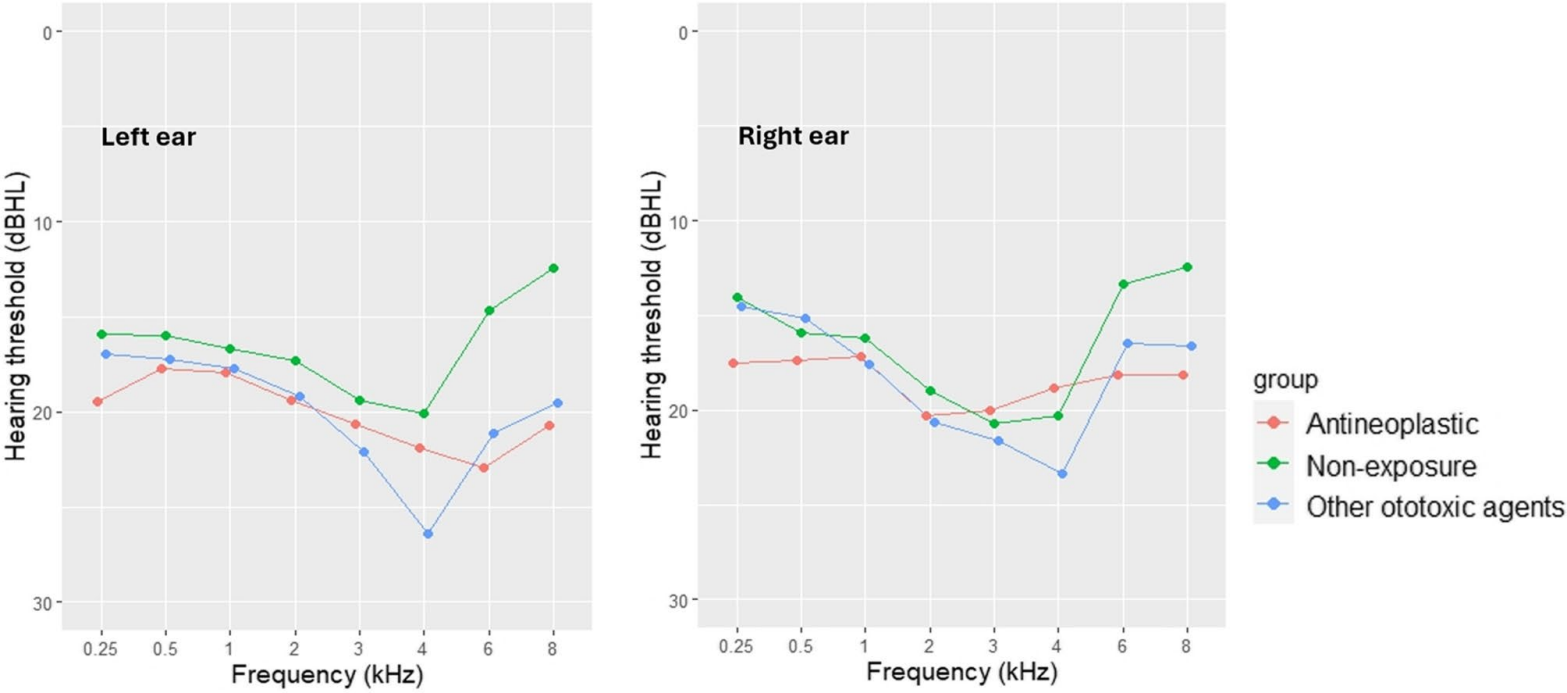
Audiograms of HCWs

The audiograms of the non-exposure and other ototoxic agent exposure groups showed bilateral notches at 4 kHz, whereas a unilateral notch at 6 kHz in the left ear was identified in the antineoplastic exposure group (Fig. 1).

In the multiple regression analysis, sex and age were significantly associated with HL (*P* < 0.05) (Table 5).

**Table 5:** Factors related to abnormal HL (*n* = 166)

	Crude OR (95%CI)	Adj. OR (95%CI)	*P* value (LR-test)
Sex (Female vs. male)	0.12 (0.03,0.4)	0.05 (0.01,0.22)	< 0.001
Age, years (cont.)	1.09 (1.04,1.14)	1.12 (1.06,1.19)	< 0.001
Obesity (Yes vs. No)	3.16 (1.35,7.36)	3.27 (1.27,8.58)	0.014

*CI* Confidence interval, *OR* Odds ratio

## Discussion

### Noise exposure levels in a hospital setting

In this study, noise exposure levels (expressed as 8-h TWA) were consistently below 85 dBA across various hospital working areas. Previous studies have primarily focused on specific departments, such as surgical suites [19–27]. The noise exposure levels in ORs (71.43 ± 4.77 dBA) were consistent with those reported by Vaisbuch et al. [28] (59.7–77.1 dBA) and Verhaert et al. [25] (72.5–76.0 dBA), both of which examined noise levels in surgical settings [26, 28]. Overall, the 8-h TWA noise exposure levels in all assessed areas remained well below the Thai and ACGIH occupational exposure limit of 85 dBA, aligning with the findings of Loupa et al. [29] but contrasting with those of other studies [3–9, 22].

### Prevalence of hearing loss

The prevalence of HL among HCWs in this study was 17.2%, which is consistent with the findings of previous studies conducted in Thailand [3–9]. Although several earlier investigations reported average noise levels exceeding 85 dBA, the noise exposure levels in the present study remained below the OELs. Furthermore, a significant proportion of participants were exposed to antineoplastic agents or co-exposed to xylene and formaldehyde, which differs from the findings of other studies [3–9]. Given that the baseline audiograms of the participants were not available, baseline audiometric assessments should be implemented among HCWs to enable more accurate identification of HL attributable to antineoplastic agents and other ototoxic agent exposure.

### Age, sex, and HL

Increasing age was associated with a higher likelihood of HL, even at 8-h TWA noise exposure levels below 80 dBA. The progression of HL was predominantly attributed to ageing and exposure to noise levels between 85 and 90 dBA among individuals aged 27–65 years [30]. No significant association was observed between NIHL and age-related HL in individuals aged 70–75 years, regardless of noise exposure status [31]. Hearing decline may therefore be influenced by chemical exposure and ageing, even when noise exposure levels remain below the OELs.

### Obesity and hearing loss

Obesity was significantly associated with HL, consistent with the findings reported among automotive workers in China [32], textile workers in Iran [33], and the Japanese working population [34]. However, Shargorodsky et al. [35] reported no such association in a study of men residing in the United States. Age, sex, and obesity were associated with HL in the present study, which aligns with the findings of Kim et al. [36], who suggested that the relationship between obesity and HL may be dependent on age and gender.

### Ototoxic agents

Four ototoxic agents commonly used in hospitals—antineoplastic drugs, xylene, formaldehyde, and mercury—were identified as potential risks to the hearing health of HCWs. This finding aligns with a report of Rai et al. [37], who classified p-xylene as a primary ototoxic agent, with a 44.6% prevalence of exposure among Australian HCWs [37]. Although both studies reported ototoxic exposures among HCWs, their contexts differed significantly. Rai et al. [37] assessed exposures from all HCW-related tasks, including patient home care and ambulance transfers. Contrarily, the present study specifically examined exposures arising from hospital-based tasks, particularly in medical laboratories and during therapeutic procedures. Mercury remains a prevalent neurotoxic and ototoxic agent in various industries, hospitals, and healthcare professionals [37–39].

Antineoplastic drugs—vincristine, doxorubicin, gemcitabine, cyclophosphamide, farmorubicin, oxaliplatin, carboplatin, and cisplatin—are ototoxic to patients undergoing chemotherapy, causing hearing loss, tinnitus, and vertigo [40, 41]. Fernandes et al. [42] reported audiometric notches in 75.75% of nurses and pharmacists exposed to chemotherapeutic agents. Contrarily, the present study demonstrated a substantially lower prevalence of 16.7% among HCWs exposed to antineoplastic agents [42]. This difference may reflect variation in the intensity and magnitude of exposure across study populations.

Xylene, particularly p-xylene, and formaldehyde are occupational chemicals with recognised ototoxic effects. These substances are also found in smoke combustion, wood manufacturing, and the painting industry [38, 43]. Animal studies have demonstrated their impact on the otorhinolaryngeal system [44]. Although generally evaluated in environmental health risk assessments for air pollutants, their specific effects within hospital settings remain underreported. Among Australian HCWs, p-xylene exposure reached 41.6% [37], higher than the prevalence observed in the present study.

Fuente, McPherson, Cardemil [45] reported associations between xylene exposure and central auditory dysfunction, even in individuals unexposed to noise levels above an 85 dBA TWA [45]. However, the health effects of combined exposure to xylene and formaldehyde in humans remain unclear despite their frequent use in pathology laboratories [46]. Animal studies have reported that the inhalation of a mixture of these compounds can lead to liver tissue damage [47]. Moreover, co-exposure to noise and formaldehyde has an additive effect on the oxidant/antioxidant system in rats [48], and formalin exposure in hospital workers has been associated with elevated levels of 15-F2t-isoprostane malondialdehyde and tumour necrosis factor α [49].

Audiometric findings in this study demonstrated bilateral 4 kHz notches in HCWs exposed to noise alone and in those with combined exposure to noise and other ototoxic agents, consistent with the pattern of occupational NIHL. A unilateral 6 kHz notch in the left ear among HCWs exposed to antineoplastic agents might indicate early cochlear toxicity associated with these agents and warrants further investigation. This finding could also reflect the tendency of antineoplastic ototoxicity to initially affect higher frequencies. The obtained results suggest a potential synergistic effect of co-exposure to noise and ototoxic agents among HCWs in this study.

To our knowledge, this study is the first study in Thailand to highlight the incidence of co-exposure to noise and ototoxic agents among HCWs despite the well-documented synergistic effects on human hearing in other industries [38]. However, a potential misclassification bias in the chemical exposure groups cannot be overlooked. Outdoor exposure to xylene during work hours, prior exposure during chemotherapy treatments and handling of antineoplastic materials or chemotherapy treatments were not captured in the self-reported questionnaire. Moreover, tasks related to transportation and patient referrals were not prioritised by ER staff. Consequently, outdoor exposure to ototoxic substances, such as xylene, during work hours may have been overlooked. Further, exposure assessments were strengthened by aligning self-reported questionnaires with annual environmental monitoring and current walkthrough surveys to define job-based exposures in this study. Future studies should integrate a task/job exposure matrix incorporating the internal biomarker levels. Extended high-frequency audiometry, distortion product otoacoustic emissions, and health questionnaires addressing ototoxic effects, such as balance disorders, should be further investigated among HCWs to clarify the aetiology of HL.

### Limitations of the study

Data on the most well-known factors affecting HL were collected. However, data on fish consumption, which could indicate exposure to mercury were not available. Non-occupational exposure to ototoxic agents was not investigated in this study. Additionally, due to constraints in laboratory equipment and budget, quantitative assessments of antineoplastic drug exposure were not performed.

## Conclusions

HL is an irreversible condition that significantly affects health and well-being. This study revealed the prevalence of HL among HCWs exposed to low noise levels and highlighted the co-exposure to noise and ototoxic agents in this population. The observed prevalence of HL among HCWs exposed to ototoxic agents highlights the importance of implementing preventive occupational measures. Since most occupational exposure limits and regulations are based on single exposures to noise or chemicals, stricter and more comprehensive programs addressing co-exposure to noise and chemicals should be enforced. Strategies, including risk assessment of HL resulting from co-exposure to noise, chemicals, and related factors, should be implemented to minimise exposure. Future investigations should focus on the effects of co-exposure to xylene and formaldehyde. Periodic audiometric testing remains essential for the early detection and prevention of HL progression among HCWs.

## Supplementary Information

Supplementary Material 1

## Data Availability

The datasets used and/or analysed during the current study are available from the corresponding author on reasonable request.

## Abbreviations

BMI: Body mass index
dB: Decibel
dBA: Decibel A
HCWs: Healthcare workers
HL: Hearing loss
HPD: Hearing protective devices
NIHL: Noise-induced hearing loss
OELs: Occupational exposure limits
OR: Odds ratio
TWA: Time-weighted average
