# Supplementary material for: Impact of ototoxic agents and noise exposure on hearing loss among healthcare personnel in a medical university: a cross-sectional analytical study
Source: BMC Public Health. 2025 Nov 20;25:4069. doi: 10.1186/s12889-025-25371-8 (PMC12632095; doi:10.1186/s12889-025-25371-8)
Supplement: Supplementary file 1 — Supplementary Material 1 [file 12889_2025_25371_MOESM1_ESM.pdf]

Pre-Screening History Form for Audiometric Assessment

Research Project: Assessment of Occupational noise exposure, noise induced hearing loss prevalence and associated factors among healthcare personnel in Medical University

HN ....

No.	Question	For researcher
1.	Age .... year	
2.	Sex ☐ Male ☐ Female ☐ Not specified	
3.	Marital status ☐ Single ☐ Married ☐ Widowed/ Divorced/ Separated	
4.	Religion ☐ Buddhism ☐ Islam ☐ Christianity ☐ Not specified	
5.	Current Workplace/ Department/ Unit (please specify)<br>..	
6.	Current Position (please specify)<br>..	
7.	Duration in Current Position (please specify) .... years/ .... months	
8.	Risk of Occupational Hearing Loss<br>8.1 Working hour exposed to loud noise per day (please specify) .... hours/day<br>8.2 Types of occupational noise exposure (check all that apply)<br>☐ Monitor alarms ☐ Machinery noise ☐ Air Conditioning noise<br>☐ Children crying ☐ Others (please specify) ....<br>8.3 Do you work with any of the following chemicals or groups of chemicals?<br>(check all that apply)<br>☐ Xylene ☐ Formaldehyde ☐ Lead ☐ Mercury ☐ Chemotherapeutic drugs	
9.	During your work, do you use hearing protection devices?<br>☐ No ☐ Yes (please specify)	

No.	Question	For researcher
10.	History of Noise Exposure<br>10.1 Have you ever worked in an environment with noise levels greater than 85 dBA (or a workplace where shouting is required for communication)?<br>☐ No ☐ Yes (please specify type of work and years of employment)<br>..<br>10.2 Do you experience loud noise exposure outside of your regular work (e.g., frequent visits to entertainment venues, woodworking, playing music, etc.)? ☐ No ☐ Yes<br>10.3 Do you use headphones/earphones for listening to music or phone calls? ☐ No ☐ Yes	
11.	**Past Medical History Related to Ear Disorders**<br>- Ear surgery ☐ No ☐ Yes<br>- Chronic suppurative otitis media ☐ No ☐ Yes<br>- Head and ear trauma ☐ No ☐ Yes<br>- Use of ototoxic medications ☐ No ☐ Yes<br>- Pre-employment sensorineural hearing loss ☐ No ☐ Yes<br>- History of acute or intense noise exposure (e.g., firecrackers, firearms, explosions, fireworks) ☐ No ☐ Yes	
12.	**Current Medical History Related to Ear Disorders**<br>- Tinnitus (ringing in the ears) ☐ No ☐ Yes<br>- Common cold, nasal congestion, ear fullness, ear pain ☐ No ☐ Yes<br>- Ear discharge (watery or purulent) ☐ No ☐ Yes	
13.	**History of Medication Use Affecting Hearing <u>(Please review medications you are currently taking or have taken in the past, if any)</u>**<br>- Antibiotics (aminoglycosides, ampicillin, macrolides), oral or ear drops ☐ No ☐ Yes<br>- Loop diuretics ☐ No ☐ Yes<br>- Antimalarial drugs (quinine, chloroquine) ☐ No ☐ Yes	

No.	Question	For researcher
	- Antineoplastic drugs (chemotherapeutic agents) ☐ No ☐ Yes<br>- Analgesics/ Anti-inflammatory drugs (aspirin/salicylates, paracetamol, codeine, indomethacin, ibuprofen, phenylbutazone)) ☐ No ☐ Yes<br>- Tetanus antitoxin ☐ No ☐ Yes<br>- Antiseptics (chlorhexidine, povidone-iodine, alcohol) ☐ No ☐ Yes	
14.	History of Alcohol, Caffeine, and Tobacco Consumption<br>14.1 Alcohol consumption ☐ Never ☐ Former drinker ☐ Current drinker<br>14.1 Caffeine consumption ☐ Never ☐ Former consumer ☐ Current consumer<br>14.1 Tobacco consumption ☐ Never ☐ Former smoker ☐ Current smoker	
15.	Before hearing test, have you been exposed to loud noise within the past 12 hours? ☐ No ☐ Yes	
